# Treatment of Depression-Related Circadian Rhythm Sleep-Wake Disorder (CRSWD) With Melatonin Receptor Agonist Ramelteon: A Case Report

**DOI:** 10.7759/cureus.68311

**Published:** 2024-08-31

**Authors:** Shuji Matsumoto, Rintaro Ohama, Takashi Hoei, Ryuji Tojo, Toshihiro Nakamura

**Affiliations:** 1 Center for Medical Science, Ibaraki Prefectural University of Health Sciences, Ami, JPN; 2 Department of Rehabilitation Medicine, Ibaraki Prefectural University of Health Sciences Hospital, Ami, JPN; 3 Department of Rehabilitation and Physical Medicine, Kagoshima University Graduate School of Medical and Dental Sciences, Kagoshima City, JPN; 4 Department of Rehabilitation, Kagoshima University Hospital, Kagoshima City, JPN; 5 Department of Rehabilitation, Acras Central Hospital, Kagoshima City, JPN

**Keywords:** rehabilitation, ramelteon, social dysfunction, melatonin, delayed sleep-wake phase disorder, circadian rhythm sleep-wake disorder, depression, insomnia

## Abstract

Insomnia, also called sleeplessness, is a sleep disorder with very diverse sleep problems and is classified into seven categories. Circadian rhythm sleep-wake disorder (CRSWD) is a type of insomnia characterized by the misalignment of the body's circadian clock with the external 24-hour environmental cycle. CRSWD encompasses seven subtypes, among which delayed sleep-wake phase disorder (DSWPD) is prominently recognized for its impact on sleep patterns. Sleep disturbances, particularly insomnia, are prevalent in depressed patients, often serving as a primary symptom that prompts clinical consultation. CRSWD frequently leads to significant social dysfunction, often making it impossible for students to attend school and difficult for working adults to find employment. Effective treatments for CRSWD include bright light therapy, cognitive-behavioral therapy for insomnia (CBT-I), and melatonin receptor agonists, particularly for certain CRSWD subtypes. In this case report, the melatonin receptor agonist ramelteon was administered to a high school student with DSWPD and comorbid depression, resulting in the successful management of symptoms. Following treatment, the patient resumed high school, pursued a university education, and secured employment post-graduation. These findings indicate that ramelteon may be a promising treatment option for CRSWD in patients with comorbid depression, warranting further clinical investigation.

## Introduction

Insomnia is defined as a type of sleep disorder in which the quality or quantity of sleep is problematic and interferes with daytime activities [1]. There are four types of insomnia: difficulty falling asleep, nocturnal awakenings, early morning awakenings, and problems falling asleep soundly. Insomnia is a prevalent symptom in depressed patients, with sleep disturbances occurring in approximately 80% of cases, as supported by recent studies [2,3]. Sleep disturbances associated with depression are varied, including difficulty initiating sleep, frequent nocturnal awakenings, early morning awakenings, and non-restorative sleep [4]. Insomnia is easily recognized and readily consulted, and many depressed patients visit clinics with sleep disturbance as a complaint [5]. According to the International Classification of Sleep Disorders, Third Edition (ICSD-3), sleep disorders are classified into seven categories, including circadian rhythm sleep-wake disorders (CRSWD), which is the focus of this review [6]. One of these sleep disorders, circadian rhythm sleep-wake disorder (CRSWD), is a sleep disorder caused by the inability of the body's internal clock to properly synchronize its cycle with the 24-hour cycle of the outside world. CRSWD is often accompanied by significant impairment of social functioning, making it difficult for students to attend school and difficult for working adults to obtain employment [1,7]. The ICSD-3 defines the diagnostic criteria for CRSWD as a discrepancy between the sleep-wake pattern and the social schedule, which causes social, occupational, and lifestyle disturbances, and the sleep-wake pattern and circadian rhythm phase are in agreement [6].

Bright light therapy and melatonin (5-methoxy-N-acetyltryptamine) agonists and rehabilitation, including cognitive-behavioral therapy for insomnia (CBT-I), are effective in advancing or retarding the circadian rhythm phase, which is important in the treatment of CRSWD [8]. Ramelteon, a melatonin receptor agonist, binds to melatonin receptor 1A (MT1) and 1B (MT2) receptors in the suprachiasmatic nucleus to regulate sleep-wake rhythms and promote sleep independent of sedation [9]. Ramelteon is also characterized by the absence of side effects such as rebound insomnia and withdrawal syndrome [10]. Because the efficacy and safety of ramelteon have not been established in patients with pre-existing or concomitant depression, we treat patients with ramelteon through frequent outpatient visits to monitor their progress and ensure that their depressive symptoms do not worsen [11].

We report here a case of CRSWD complicated by depression treated with ramelteon. After treatment with ramelteon, the patient returned to high school, enrolled in and graduated from college, and subsequently found employment and improved social dysfunction.

## Case presentation

**Figure 1:**
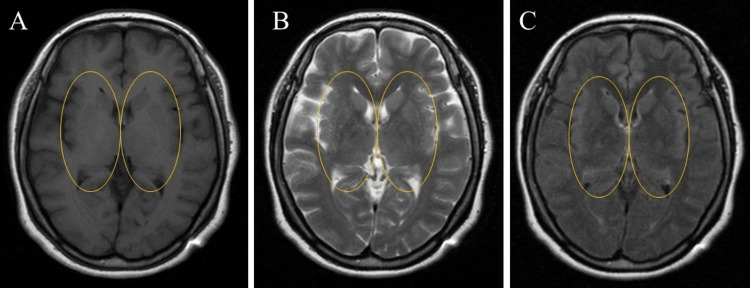
Brain MRI at the level of the basal ganglia Brain MRI findings were normal. There are no abnormalities in the basal ganglia (striatum, globus pallidus, subthalamic nucleus, substantia nigra), thalamus, or posterior limb of the internal capsule (circled areas). A: T1 weighted image; B: T2 weighted image; C: fluid attenuated inversion recovery (FLAIR) image

The patient is a 22-year-old female who has been treated as an outpatient for the past five years. She had bronchial asthma since the age of five years. The current medical history shows the onset of a sleep disorder at the age of 14 years, with difficulty falling asleep and shortened sleep time due to awakening in the middle of the night. With the onset of the sleep disorder, the patient gradually developed anorexia, weight loss, decreased motivation and interest, depressed mood, and a sense of sadness. She began refusing to go to school at age 16 and was unable to complete her high school credits due to a lack of attendance. At age 17, she made her first visit to our outpatient clinic in February 2019. Magnetic resonance imaging (MRI) (Figure 1) of the head, blood biochemistry, and thyroid function tests were normal, and physical examination findings were also normal. Utilizing the Diagnostic and Statistical Manual of Mental Disorders (DSM)-5-TR diagnostic criteria [12], the patient was diagnosed with severe major depressive disorder, characterized by decreased motivation, anhedonia, persistent depressed mood, and sadness persisting for over two years.

The patient’s sleep pattern was marked by delayed sleep onset, typically around 2:00 a.m., followed by multiple nocturnal awakenings and waking up at approximately 10:00 a.m. The patient was very hopeful to somehow graduate from high school and also wanted to improve her sleep disorder, so she was started on fluvoxamine 50 mg/day and ethyl loflazepate 2 mg/day in March 2019. After one month of oral administration, there was no improvement, and instead, she began to have nightmares (being chased by black butterflies, rabbits jumping up and down, etc.), which were accompanied by suicidal thoughts, so in April 2019, trazodone 50 mg/day was added. Although the patient improved to the point where she was able to attend high school, the nightmares did not disappear. Due to the persistence of symptoms, fluvoxamine and ethyl loflazepate were discontinued in August 2019, and the patient was transitioned to duloxetine 40 mg/day to better manage her depressive and anxiety symptoms. Her appetite increased, her depressed mood improved, her motivation improved, and she was able to work part-time, but her sleep disorder did not improve.

**Figure 2:**
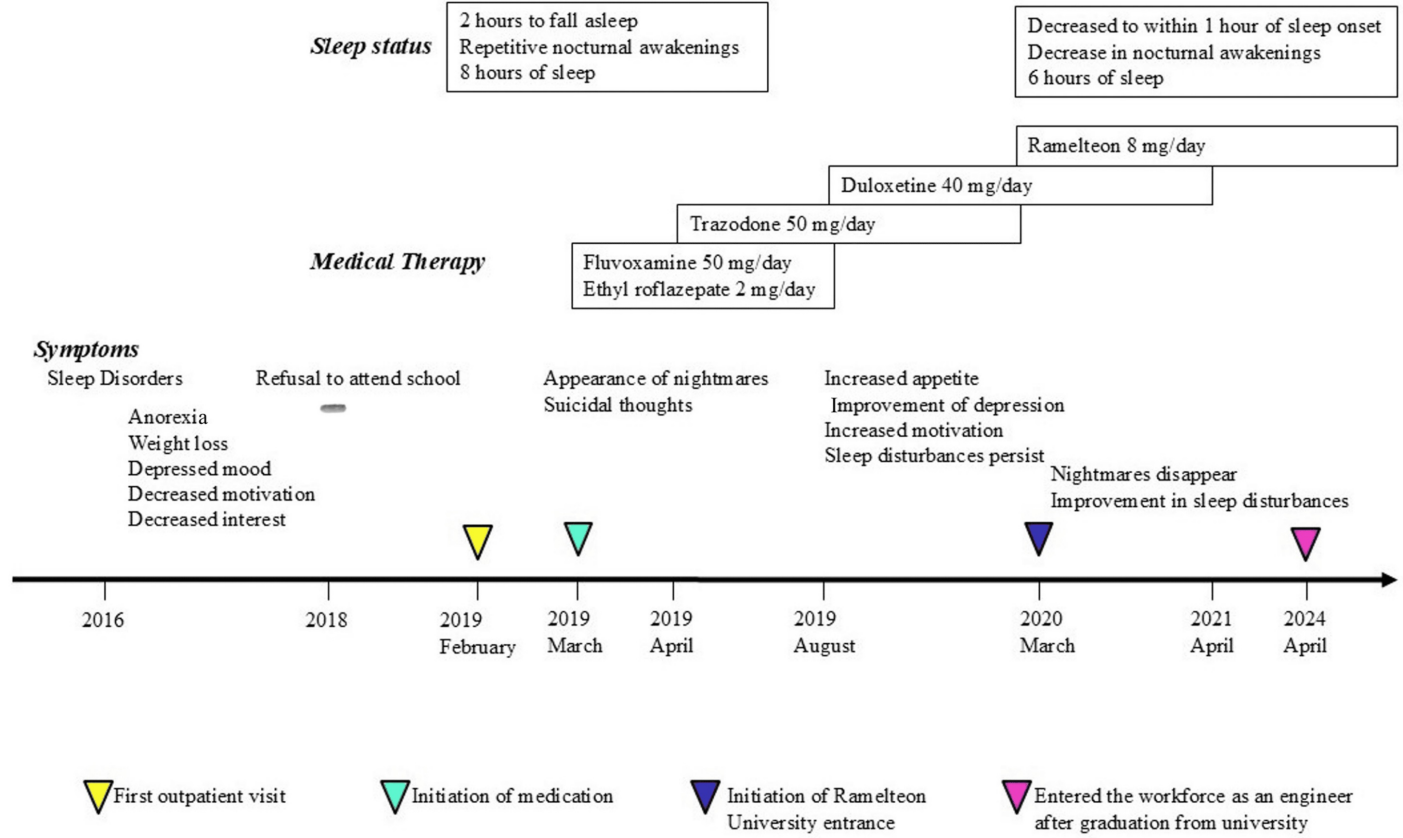
Clinical course of the case Sleep status is shown in the upper panel, medications in the middle panel, and symptoms over time in the lower panel.

She continued to have nightmares, and there was a discrepancy between her sleep-wake pattern and her social life schedule, which was causing social, occupational, and lifestyle problems, meeting the diagnostic criteria for CRSD. In March 2020, trazodone was discontinued and ramelteon 8 mg/day was started. The patient was instructed to take ramelteon around 10 p.m. and to go to bed at 11 p.m. After about two weeks, her nightmares disappeared, her sleep disorder improved, she was able to fall asleep by midnight, her nighttime awakenings decreased, and her waking pattern changed to around 6 a.m. In April 2021, duloxetine was also discontinued, and the patient continues to take ramelteon 8 m/day only, with good sleep rhythm and disappearance of depressive symptoms (Figure 2). The patient graduated from high school in March 2020, entered the technical university in the same year, graduated from the university in March 2024, and started working as an engineer this year. As of August 2024, she continues to visit the outpatient clinic once a month.

## Discussion

Sleep disorders are prevalent across various medical specialties due to their high incidence in the general population, particularly among individuals with comorbid conditions such as depression [5]. Sleep disturbances are one of the most commonly reported symptoms among patients with depression, often serving as a primary reason for seeking medical attention. In fact, many patients who complain of sleep disturbances are found to have depression as a complication [2]. Cohort studies of depression and sleep disorders have demonstrated a high odds ratio for depression and sleep disorders [13]. According to the ICSD-3, sleep disorders are categorized into seven groups, with CRSWD being particularly relevant in the context of comorbid depression [6]. Among these sleep disorders, CRSWD refers to sleep disorders caused by the inability of the body to properly synchronize its biological clock with the 24-hour cycle of the outside world. As highlighted by Kamei et al. [14], CRSWD frequently leads to substantial social dysfunction, as evidenced in this case, where the patient's academic performance and future employment prospects were severely impacted. The diagnostic criteria for CRSWD are a discrepancy between the sleep-wake pattern and the social schedule, which causes social, occupational, and lifestyle disturbances, and the sleep-wake pattern and circadian rhythm phase should be in agreement. Tagaya et al. [8] stated that the second criterion is an objective indicator and that if it is satisfied, it is safe to conclude that there is an abnormality in the circadian rhythm mechanism. It is noteworthy that even in healthy individuals, temporary disruptions in sleep patterns, such as those occurring during vacations, can lead to transient CRSWD-like symptoms, underscoring the vulnerability of the circadian system to external influences. This suggests that the presence of abnormal sleep-wake patterns and circadian rhythm phase coincidence does not indicate abnormalities in the circadian rhythm mechanism. A delayed sleep-wake pattern with excessively late sleeping and late awakening is common, especially when social schedule enforcement is compromised by non-attendance or absence from school. It has been reported that it is difficult to determine whether this is caused by an abnormal circadian rhythm mechanism, whether the patient simply chooses this type of lifestyle pattern, or whether there are other causes, which also makes the diagnosis of CRSWD difficult [8].

The ICSD-3 classifies CRSWD into two types associated with artificial schedule changes (time-lag and shift work) and seven types that appear without artificial schedule changes (sleep-phase regression, sleep-phase advance, irregular sleep-wake, free-again type, CRSWD due to physical illness, drug- or substance-related CRSWD, and other CRSWD). Tagaya et al. [8] classified depression and depressive state in CRSWD into four types: time-lagging type, shift work type, sleep-phase regressive type, and free-again type. In this classification, the case reported here is considered to fall into the sleep-phase regressive type. It is important to note that the treatment of CRSWD involves either forward or backward circadian rhythm phases.

Bright light therapy, melatonin supplementation, and melatonin receptor agonists like ramelteon are effective in modifying the circadian rhythm, either by advancing or delaying the sleep phase, thereby addressing the underlying circadian misalignment in CRSWD [8]. Exposure to bright light when the body clock is in the evening or midnight phase (several hours before and after falling asleep the previous day) causes the body clock to go backward, while exposure to bright light when the body clock is in the morning phase (several hours before and after awakening the previous day) causes the body clock to go forward [15]. A small dose of melatonin (0.25 to 1 mg) or a small dose of melatonin receptor agonist (2 to 4 mg) taken four to eight hours before the time of falling asleep on the previous day advances the biological clock, while a dose taken on awakening sets the biological clock back. High-intensity light and melatonin or melatonin agonist (agomelatine) doses are taken according to the time when sleep is emerging at that point [16,17]. Ramelteon, a melatonin receptor agonist, binds to melatonin (MT1, MT2) receptors in the suprachiasmatic nucleus and selectively acts on them. Ramelteon primarily functions by reducing sleep latency, extending total sleep duration, and advancing the sleep phase, all of which are critical in realigning the patient's circadian rhythm and improving their overall sleep quality, as demonstrated in this case. The maximum blood concentration of ramelteon is reached at 45 minutes after administration, and its elimination half-life is relatively short, ranging from one to two hours [18]. Ramelteon has no affinity for GABA-A receptors, opioid, muscarinic, serotonin, or dopamine receptors. Therefore, ramelteon does not cause side effects such as cognitive dysfunction and motor dysfunction, which have been observed with conventional sleeping pills, and there are no abuse or dependence problems. Ramelteon is a drug that regulates sleep-wake rhythms and promotes physiological sleep without sedative or anxiolytic effects. It is also characterized by the absence of side effects such as rebound insomnia and withdrawal syndrome and is therefore considered to be a safer sleep medication than other sleep medications [17,19].

In the present study, we started the patient on the same dose of ramelteon (8 mg/day) that was used in previous reports of its effectiveness in insomnia associated with depression [11,14,18]. The patient had been truanting from school due to sleep disturbance, which interfered with her schoolwork. Despite taking various antidepressants and sleep-inducing drugs, her sleep disturbance did not improve, and finally, when ramelteon was administered one hour before bedtime, her sleep improved. After taking ramelteon, the patient earned credits for graduation from high school, graduated successfully, and was even able to find a job, proving that the drug was highly effective. The patient was closely monitored through frequent outpatient visits, allowing for the timely adjustment of treatment and ensuring that her sleep disorder improved without exacerbating her depressive symptoms. However, the efficacy and safety of ramelteon in patients with pre-existing or concomitant depression have not been established, and one of the important points of ramelteon administration is that its concomitant use with the antidepressant fluvoxamine is contraindicated, so caution is required [20].

## Conclusions

This case involved a 22-year-old patient diagnosed with severe major depressive disorder according to DSM-5-TR criteria who also met the diagnostic criteria for circadian rhythm sleep-wake disorder (CRSWD) as outlined in the ICSD-3. The patient’s misalignment between her sleep-wake cycle and daily social schedule resulted in significant social, occupational, and lifestyle disruptions, highlighting the profound impact of CRSWD on quality of life. Treatment with the melatonin receptor agonist ramelteon led to significant improvements in the patient’s sleep patterns, allowing for the discontinuation of antidepressants and resulting in the resolution of her social, occupational, and lifestyle difficulties. This case suggests that with careful monitoring, ramelteon may be an effective treatment option for CRSWD in patients with comorbid depression, warranting further clinical investigation to confirm its efficacy and safety in broader populations.
